# Primary Extracranial Ewing Sarcoma Complicated by Hemorrhagic Shock and Multimicrobial Infection: A Diagnostic and Therapeutic Challenge

**DOI:** 10.7759/cureus.100568

**Published:** 2026-01-01

**Authors:** Marta Anastácio, Marta Roldão, Andreia Salgadinho Machado, Daniela Maia, Ana Lynce

**Affiliations:** 1 Internal Medicine, Centro Hospitalar de Lisboa Ocidental, Lisbon, PRT; 2 Internal Medicine, Hospital São Francisco Xavier, Lisbon, PRT; 3 Hematology, Instituto Português de Oncologia de Lisboa Francisco Gentil, Lisbon, PRT; 4 Internal Medicine Departement, Centro Hospitalar de Lisboa Ocidental, Lisbon, PRT

**Keywords:** ewing sarcoma, ewsr1 rearrangement, extracranial neoplasm, polymicrobial infection, tumoral hemorrhage

## Abstract

Ewing sarcoma is a rare malignant neoplasm that is seldom located in the skull or scalp. We describe the case of a 19-year-old previously healthy man presenting with a rapidly growing left parieto-occipital exophytic mass following minor trauma, initially misinterpreted as an abscess. The lesion recurred exponentially with polymicrobial infection and progression to massive extracranial tumoral hemorrhage, culminating in hemorrhagic shock. Surgical excision and histopathological examination confirmed Ewing sarcoma with *EWSR1 *gene rearrangement. Staging revealed pulmonary, lymph node, and bone metastases. Despite the initiation of palliative chemotherapy, the patient experienced rapid tumor progression with recurrent hemorrhage, multiorgan failure, and death. This case highlights the rarity, aggressiveness, and diagnostic complexity of extracranial Ewing sarcoma, emphasizing the importance of a multidisciplinary approach and early molecular confirmation.

## Introduction

Ewing sarcoma is a rare malignant neoplasm belonging to the group of small round blue cell tumors, characterized by high aggressiveness, rapid progression, and an early tendency to metastasize [1-4]. It predominantly affects adolescents and young adults and most commonly arises in the long bones and pelvis [1,2,4]. Extracranial localization is exceptional, accounting for less than 1% of reported cases [1,2,5]. These lesions exhibit a broad differential diagnosis and may mimic meningiomas, infectious, or metastatic processes, which frequently leads to diagnostic delay [3,5-7].

Recent advances in immunohistochemistry and molecular genetics - notably the identification of the t(11;22)(q24;q12) translocation and diffuse CD99 expression - have been instrumental in distinguishing this tumor from other morphologically similar neoplasms [1,4,6,7].

Despite improvements in multimodal therapy, the prognosis remains poor, particularly in cases with metastatic disease at diagnosis or early relapse [1-4]. In addition to tumor aggressiveness, infections related to immunosuppression are a significant cause of morbidity and mortality in these patients, as are potentially fatal local complications, such as tumoral hemorrhage resulting from the high vascularity and tissue fragility associated with aggressive, ulcerated tumors [3-5,8].

We present the case of a previously healthy young adult with metastatic extracranial Ewing sarcoma, whose clinical course illustrates the diagnostic and therapeutic challenges of this rare entity and underscores the importance of early clinical suspicion in rapidly growing extracranial tumors.

## Case presentation

A 19-year-old melanodermic man from São Tomé and Príncipe, previously healthy, with no relevant family history, no chronic medication use, no known allergies, no occupational exposures, and denied tobacco, alcohol, or other toxin use, presented with a progressively enlarging swelling in the left parieto-occipital region over six months, following a minor head injury from a fall. The lesion was initially interpreted as an abscess and surgically drained at a local hospital. However, early recurrence was noted, with exponential growth, purulent discharge, and foul odor, associated with asthenia, anorexia, and unquantified weight loss (BMI 17 kg/m²; weight 48 kg; height 168 cm). The patient was admitted for investigation and later transferred to Portugal for specialized care. On admission to a Portuguese hospital, he appeared cachectic, with left facial edema, ipsilateral neck flexion, and a large, ulcerated, painful, bleeding parieto-occipital exophytic mass. Neurological examination was normal. The laboratory results are presented in Table 1.

**Table 1:** Laboratory results at hospital admission

Test	Result	Normal range
Hemoglobin (g/dL)	7.0	12.0-13.0
Mean corpuscular volume (fL)	76	80-60
Mean corpuscular hemoglobin concentration (g/dL)	33	32-36
Leukocytes (x 10⁹/L)	15.7	4.0-10.0
Neutrophils (%)	88.5	40-80
C-reactive protein (mg/dL)	9.12	<0.5
Albumin (g/dL)	1.5	3.5-5.5

**Figure 1:**
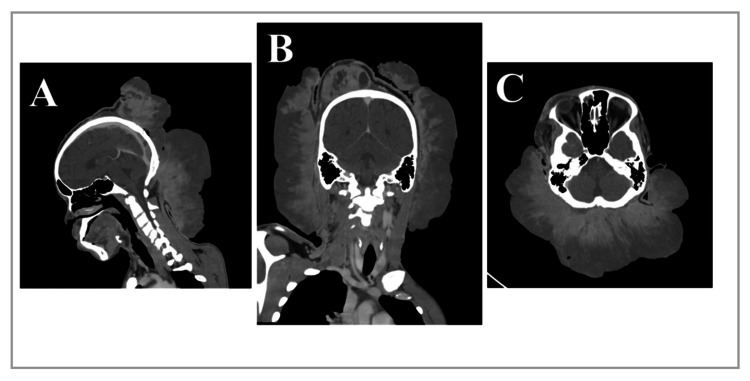
Cranial CT scan images showing a large and exophytic left parieto-occipital mass: sagittal (A), coronal (B), and axial (C) reconstructions CT: Computed tomography.

Cranioencephalic CT (Figure 1) scan revealed an extracranial mass with epidural extension and bone invasion. Thoracoabdominopelvic CT showed bilateral pulmonary micronodules up to 6 mm, hepatomegaly, and hilar lymphadenopathy (15 mm). Due to symptomatic anemia, he received three packed red blood cell transfusions with poor response (final hemoglobin 8.3 g/dL).

Blood cultures (aerobic and anaerobic) were collected, and empiric piperacillin-tazobactam 4.5 g every six hours was initiated for suspected bacterial infection and risk of *Pseudomonas aeruginosa* associated with prolonged hospitalization. Superficial biopsy of the extracranial mass revealed dense inflammatory infiltrate without neoplastic cells. Immunohistochemistry was negative for carcinoma (AE1/AE3), HHV-8, and fungi (Grocott stain). Bacterial, fungal, and parasitic cultures, as well as PCR for *Mycobacterium tuberculosis*, nontuberculous mycobacteria, and pan-fungal targets, were negative, as was Leishmania testing. Immunophenotyping could not be performed due to insufficient cellular material, and flow cytometry results were normal. Given the absence of microbiologic growth and persistent inflammation, piperacillin-tazobactam was discontinued after 16 days, and an antibiotic-free interval was maintained. During hospitalization, the patient developed a diffuse and severe headache, refractory to standard analgesia, and required opioid therapy under the supervision of the palliative care team.

**Figure 2:**
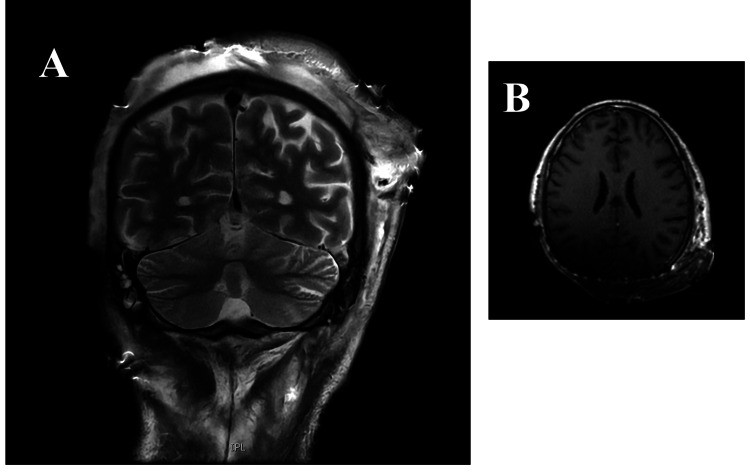
Postoperative MRI imaging of the brain: axial T1-weighted (A) and coronal T2-weighted (B) sequences after gadolinium administration showing residual enhancing lesion next to the titanium mesh in the left parieto-occipital region MRI: Magnetic resonance imaging.

Due to severe malnutrition, he was evaluated by a nutritionist and started on a high-protein, high-calorie diet. On hospital day 11, the patient experienced a sudden massive hemorrhage from the extracranial tumor, causing hemodynamic instability and a drop in hemoglobin to 2.5 g/dL, consistent with hemorrhagic shock. He was transferred to the intensive care unit (ICU) and received multiple transfusions (packed red blood cells, platelet pools, fibrinogen, and ferric carboxymaltose). Given radiologic evidence of epidural invasion, the case was discussed in a multidisciplinary meeting with plastic surgery and neurosurgery, and urgent surgery was indicated. An en bloc excision of the mass (1.6 kg) was performed, including craniectomy of the invaded area, removal of the epidural component, cranioplasty with titanium mesh, and coverage using a free flap from the latissimus dorsi and left thigh. Postoperative cranial CT showed no intracranial complications, though brain MRI (Figure 2) confirmed residual tumor compatible with incomplete (R2) resection. Postoperatively, the patient developed fever and elevated inflammatory markers (CRP 16.8 mg/dL). Following infectious disease consultation, empiric meropenem 1 g every eight hours and continuous infusion vancomycin 19 mg/50 mL at 4.2 mL/h (adjusted by serum levels) were initiated.

**Figure 3:**
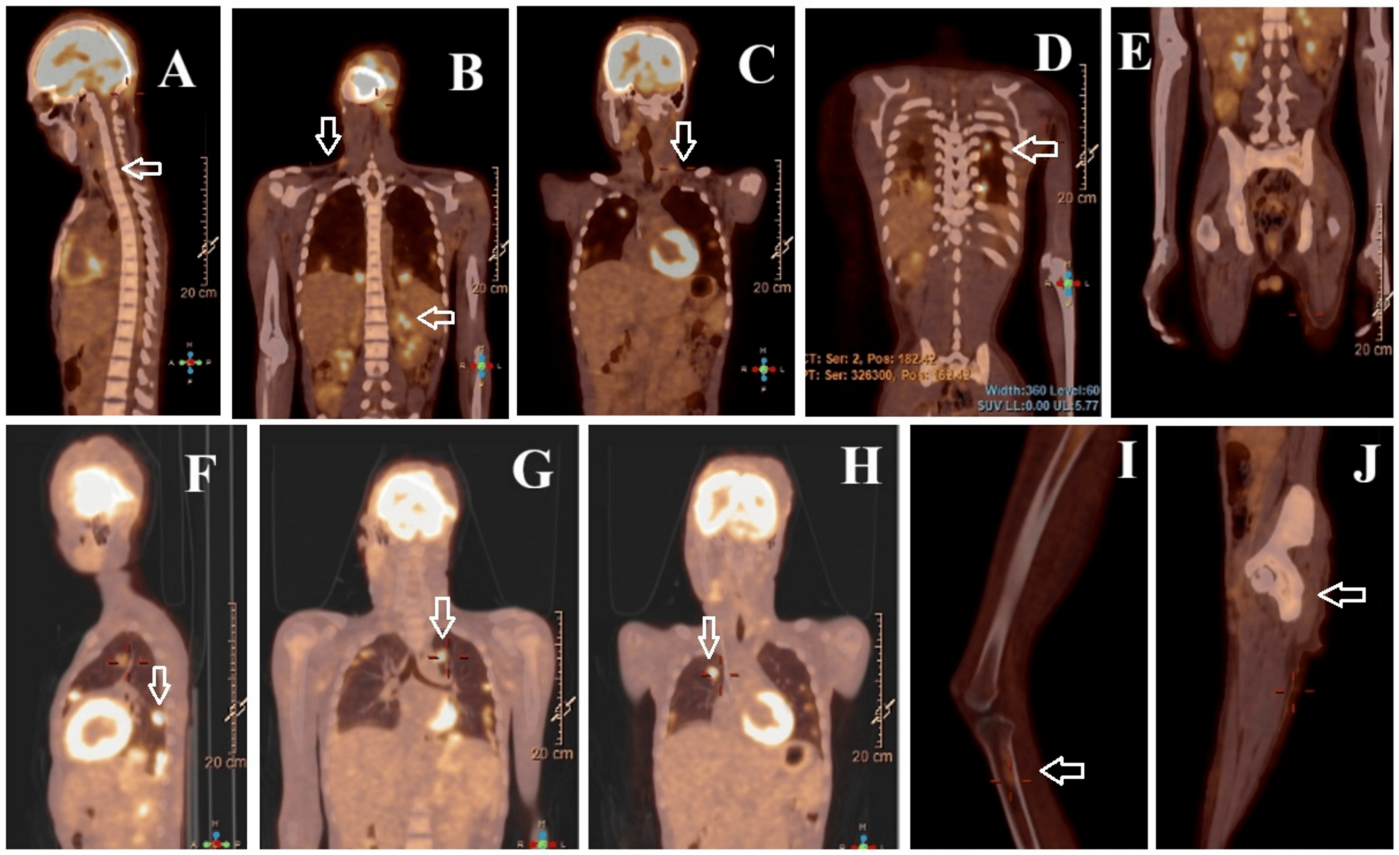
PET-CT images (coronal and sagittal views) demonstrating multiple foci of pathological FDG uptake (white arrows): (A) cervical vertebra metastasis, (B-C) cervical lymph nodes metastasis; (D-H) multiple lung metastases; (I-J) proximal right tibia metastasis FDG: Fluorodeoxyglucose; PET-CT: Positron emission tomography-computed tomography.

Culture of the excised mass revealed polymicrobial infection with *Enterococcus faecalis*, *Escherichia coli* (ESBL (extended-spectrum betalactamase)-producing, meropenem-sensitive), *Pseudomonas mendocina* (ceftolozane-tazobactam-sensitive), and *Candida duobushaemulonii*. The patient received directed meropenem, continued empiric vancomycin, and was started on ceftolozane-tazobactam 1.5 g every eight hours for 21 days and caspofungin (18 days) with a favorable clinical and laboratory response (CRP decreased to 3.7 mg/dL). Plastic surgery confirmed complete graft take and viable underlying musculature. Daily wound care was maintained. Histopathological analysis of the surgical specimen showed a lobulated, exophytic, ulcerated, necrotic tumor (7 cm) composed of small, round, blue cells. Immunohistochemistry was positive for CD99, vimentin, CD56, factor XIII, and cyclin D1, and negative for AE1/AE3, CK8/18, EMA, CD3, CD20, MPO, TdT, CD43, NSE, SOX10, chromogranin A, synaptophysin, GFAP, Melan-A, S100, CD138, desmin, AML, ERG, and WT-1. The Ki-67 proliferative index was 80%. Detection of EWSR1 gene rearrangement (22q12) confirmed the diagnosis of Ewing sarcoma. The patient was transferred to the Portuguese Oncology Institute (Instituto Português de Oncologia Francisco Gentil), where staging PET-CT (Figure 3) revealed cervical lymph node, bilateral pulmonary, and bone metastases (cervical vertebra and proximal right tibia).

Palliative chemotherapy with vincristine, cyclophosphamide, and doxorubicin was initiated, but only one cycle was completed due to rapid clinical deterioration. In the following weeks, the patient developed recurrent frontal headache, vomiting, visual deficits (visual agnosia), and fever. Neuro-ophthalmologic evaluation revealed impaired conjugate gaze to the right and bilateral papilledema without exudates. Follow-up CT and brain MRI demonstrated massive tumor expansion with hemorrhagic components, including invasion of the superior sagittal sinus, transverse sinus, right occipital lobe (6 × 4 cm), posterior superior infiltration of the right cerebellar hemisphere (2 cm), with cerebral edema and midline shift, as well as another large parieto-occipital mass (15 × 7 cm). Despite intensive management (dexamethasone up to 24 mg/day, mannitol, acetazolamide 250 mg every eight hours, antiepileptic prophylaxis, and major analgesia), the patient remained refractory, with progressive neurological and systemic deterioration. After multidisciplinary discussion, systemic oncologic treatment was discontinued, and exclusive palliative care was pursued. A single palliative radiotherapy session (8 Gy) was attempted but not tolerated. The patient developed recurrent tumoral hemorrhage, progressive loss of consciousness, and terminal respiratory failure, ultimately resulting in death. A timeline summarizing disease progression and development is presented in Table 2.

**Table 2:** Timeline summary of disease progression and development CT: Computed tomography; IHC: Immunohistochemistry; MRI: Magnetic resonance imaging; PET-CT: Positron emission tomography-computed tomography; VDC: Vincristine, cyclophosphamide, and doxorubicin; ESBL: Extended-spectrum betalactamase.

Timeline	Clinical events	Key findings/Diagnostic studies	Therapeutic interventions
At presentation	Progressively enlarging left parieto-occipital swelling after minor trauma; recurrence with purulent discharge, with weight loss	—	—
6 months after - hospital admission in São Tomé and transfer to Portugal	Cachexia, facial edema, and ulcerated parieto-occipital exophytic mass	CT: extracranial mass with epidural extension, and bone invasion; complete body CT: pulmonary micronodules, hepatomegaly, and hilar adenopathy	—
First week after admission to a Portuguese hospital	Symptomatic anemia	—	Transfusion support
Hospital day 11 (Hemorrhagic shock)	Massive extracranial tumor bleeding	Hemoglobin drops until 2.5 g/dL	ICU admission and transfusion support
Hospital day 11 (Urgent surgery)	Decision for en bloc excision	—	Tumor excision, craniectomy, and cranioplasty
Hospital day 12 (Postoperative)	Fever, elevated C-reactive protein	Mass infection	Started meropenem and vancomycin
Hospital day 18 (Microbiologic diagnosis)	Polymicrobial mass infection	Isolation of *E. faecalis*, *E. coli* (ESBL), *P. mendocina*, *C. duobushaemulonii*	Added ceftolozane-tazobactam and caspofungin
Hospital day 27 (Histopathological diagnosis and molecular biology)	—	Histopathology with small round blue cell tumor; IHC positive for CD99, vimentin, CD56; EWSR1 rearrangement compatible with Ewing sarcoma	Referred and transferred to oncology
Hospital day 30 (Staging PET-CT)	—	Metastatic disease: cervical nodes, lungs, cervical vertebra, and right tibia	—
Hospital day 45 (Oncology unit)	Clinical deterioration	—	1 cycle VDC chemotherapy
Hospital day 48 (Neurological decline)	Headache, visual loss, and papilledema	MRI: massive tumor expansion and sinus invasion	Dexamethasone, mannitol, acetazolamide, and analgesia
Hospital day 65 (Palliative phase)	Refractory symptoms	—	Palliative radiotherapy (not tolerated)
From hospital day 67 until death	Recurrent tumoral hemorrhage, coma, and respiratory failure	—	Palliative care

## Discussion

Extracranial Ewing sarcoma represents one of the rarest and most aggressive forms of primitive neuroectodermal tumors, accounting for less than 1% of reported cases of extraskeletal Ewing sarcoma [1,2,4]. The diagnostic challenge arises not only from its low incidence but also from its morphologic and radiologic resemblance to other extracranial lesions - benign, malignant, or infectious [3,5-7]. Clinical clues that should raise awareness for cranial Ewing sarcoma include presentation in children or young adults, rapidly progressive neurological symptoms, refractory headache, focal deficits or seizures, and radiologic evidence of an aggressive skull-based, calvarial, or extra-axial lesion with osteolytic changes and soft tissue invasion. In several reports, such masses were initially misinterpreted as meningiomas, metastases, or abscesses, with the definitive diagnosis frequently delayed until histopathologic and immunohistochemical confirmation [5-7]. The present case reinforces this pattern: the superficial biopsy revealed only necrosis and inflammatory infiltrate - a limitation also observed by Kumarasamy et al. (2024), who reported inconclusive results in approximately one-third of initial samples due to extensive tumor necrosis [1].

In recent years, genetic advances have redefined diagnostic precision. Studies such as Kim et al. (2024) demonstrated that atypical molecular signatures, including uncommon EWSR1 gene rearrangements, may alter the immunophenotypic profile, requiring complementary in fluorescent in situ hybridization and sequencing-based techniques for definitive identification [4,6,7]. These developments highlight the importance of close collaboration among pathology, molecular genetics, and oncology teams for accurate diagnosis, while future perspectives for earlier detection rely on increased clinical awareness and the early application of molecular diagnostics, including fluorescence in situ hybridization or next-generation sequencing to identify EWSR1 rearrangements, particularly in limited or non-diagnostic biopsy specimens [1,4,6].

The biological aggressiveness of extracranial Ewing sarcoma often results in rapid progression, early metastasis, and local recurrence, with overall survival rates below 24 months even under multimodal therapy [1-4]. The literature documents atypical presentations, including cranial nerve palsies and diffuse epidural invasion, reflecting the phenotypic heterogeneity of this entity [2,3]. In the present case, massive extracranial tumoral hemorrhage was the predominant complication - a rarely reported but plausible manifestation in highly vascular, centrally necrotic tumors [1,3,5]. Pandey et al. (2025) emphasized that aberrant vascularization of extracranial lesions may precipitate acute hemorrhagic events with fatal outcomes, particularly in cases involving dural invasion and epidural compromise [5]. This phenomenon underscores the hemostatic fragility and dynamic instability of the tumor microenvironment in such neoplasms [1,3,5].

The clinical course of this patient was further complicated by severe polymicrobial infections, an increasingly recognized issue among immunocompromised oncology patients [8]. The isolation of *E. faecalis*, ESBL-producing *E. coli*, *P. mendocina*, and *C. duobushaemulonii* - a recently described multidrug-resistant member of the *Candida haemulonii* complex - illustrates the growing diversity of opportunistic pathogens in this context [9,8,10-13].* C. duobushaemulonii* is often misidentified as *Candida albicans* in automated systems, leading to potential therapeutic mismanagement [11,13]. Molecular characterization is therefore essential for accurate identification and selection of appropriate antifungal therapy, as this species may exhibit partial resistance to echinocandins [9,10-12]. These findings reinforce the increasing role of precision microbiology in the management of complex infections in oncology patients [8,9,10-13].

Therapeutic decision-making in this case exemplified the need for a truly multidisciplinary approach, involving neurosurgery, plastic surgery, infectious diseases, oncology, and palliative care [1,2,4]. Multimodal management should integrate radical surgical resection, systemic chemotherapy, and radiotherapy, preferably within specialized centers [1,2,4]. However, in patients presenting with widespread metastases or early relapse, the therapeutic goal often becomes palliative, focusing on symptom control and quality of life [1-4]. The rapid clinical deterioration observed in this patient reflects the fulminant nature of the disease and the limitations of current interventions. Despite advances in multimodal treatment, therapeutic management of extracranial Ewing sarcoma remains particularly challenging, especially in patients with aggressive clinical presentations. Standard systemic chemotherapy regimens, such as vincristine-doxorubicin-cyclophosphamide alternating with ifosfamide-etoposide, may be limited by significant toxicity and reduced efficacy in cases with early relapse or disseminated disease. Similarly, the role of radiotherapy is constrained by the proximity of critical neural structures, requiring careful dose balancing to achieve local control while minimizing neurotoxicity. In rapidly progressive cases, clinical deterioration may significantly narrow the therapeutic window, precluding timely initiation or completion of intensive treatments and shifting the therapeutic focus toward palliative care [1-4].

Emerging targeted therapeutic strategies for Ewing sarcoma include agents directed against the EWSR1-FLI1 oncogenic pathway, insulin-like growth factor 1 receptor (IGF-1R) inhibitors, poly(ADP-ribose) polymerase (PARP) inhibitors, and cell-cycle-targeted therapies, although their clinical benefit remains under investigation. To date, immunotherapy has shown limited efficacy, underscoring the need for continued research into molecularly driven and precision-based treatments.

This case illustrates, in a paradigmatic manner, three key dimensions of modern clinical practice: the diagnostic complexity of small round blue cell tumors in extracranial locations, the infectious and hemorrhagic vulnerability associated with tumor biology and immunosuppression, and the need for early integration of multidisciplinary and precision medicine approaches. Early recognition of these distinctive features is crucial to optimize diagnosis, prevent complications, and guide individualized therapeutic decision-making in tumors of such aggressive and unpredictable behavior.

## Conclusions

Extracranial Ewing sarcoma is a rare and highly aggressive entity whose atypical presentation continues to pose a major diagnostic challenge. Its clinical and radiologic resemblance to meningiomas, metastases, or infectious processes can delay recognition and compromise early management. Diagnosis requires close correlation among pathology, molecular genetics, and imaging, with identification of specific markers such as CD99 expression and EWSR1 gene rearrangements. The simultaneous occurrence of massive extracranial tumor bleeding and severe polymicrobial infections in this case illustrates both the aggressiveness and vulnerability associated with this neoplasm. Early clinical suspicion is therefore crucial when evaluating rapidly growing extracranial tumors in young patients. This case underscores the critical role of multidisciplinary collaboration in complex decision-making and highlights the need for an integrated management strategy, with individualized treatment and early palliative support in metastatic cases. The reporting of rare cases such as this contributes to expanding current knowledge regarding unusual presentations, severe complications, and adaptive therapeutic strategies in extracranial Ewing sarcoma.
